# Validation of a Loop-Mediated Isothermal Amplification-Based Kit for the Detection of *Legionella pneumophila* in Environmental Samples According to ISO/TS 12869:2012

**DOI:** 10.3390/microorganisms12050961

**Published:** 2024-05-10

**Authors:** Giorgia Caruso, Maria Anna Coniglio, Pasqualina Laganà, Teresa Fasciana, Giuseppe Arcoleo, Ignazio Arrigo, Paola Di Carlo, Mario Palermo, Anna Giammanco

**Affiliations:** 1 U.O.C. of Microbiology and Virology, ARNAS “Civico Di Cristina and Benfratelli”, 90127 Palermo, Italy; 2 Legionella Reference Laboratory, Department of Medical, Surgical Sciences and Advanced Technologies “G.F. Ingrassia”, University of Catania, Via Santa Sofia 87, 95123 Catania, Italy; maconi@unict.it; 3 Hygiene Complex Operative Unit, A.O.U. Policlinico—Vittorio Emanuele, Via S. Sofia 87, 95123 Catania, Italy; 4 Legionella Reference Laboratory, University of Messina, 98125 Messina, Italy; pasqualina.lagana@unime.it; 5 Department of Biomedical and Dental Sciences and Morphofunctional Imaging, University of Messina, 98125 Messina, Italy; 6 Legionella Reference Laboratory, University of Palermo, 90127 Palermo, Italy; ignazio.arrigo90@gmail.com (I.A.); anna.giammanco@unipa.it (A.G.); 7 Department of Health Promotion, Mother and Child Care, Internal Medicine and Medical Specialties, University of Palermo, 90127 Palermo, Italy; paola.dicarlo@unipa.it; 8 Enbiotech s.r.l., Via Aquileia 34, 90144 Palermo, Italy; gg.arcoleo@enbiotech.eu; 9 Sicilian Health Department, Public Health and Environmental Risks Service, 90127 Palermo, Italy; mario.palermo1955@gmail.com

**Keywords:** *L. pneumophila*, LAMP, environmental samples, rapid identification

## Abstract

*Legionella pneumophila* is a freshwater opportunistic pathogen and the leading cause of severe pneumonia known as Legionnaires’ disease. It can be found in all water systems and survives in biofilms, free-living amoebae, and a wide variety of facilities, such as air conditioning and showers in hospitals, hotels and spas. The reference cultural method allows for the isolation and identification in many days, and in addition, it does not detect viable but rather non-culturable bacteria, increasing the risk of infection. In this context, a new LAMP-based (loop-mediated isothermal amplification) kit was developed, allowing for the rapid, sensitive, and labor-saving detection of *L. pneumophila*. The kit, “*Legionella pneumophila* Glow”, was validated according to ISO/TS 12869:2012, testing sensitivity, inclusivity and exclusivity, and kit robustness. Sensitivity showed that the “*Legionella pneumophila* Glow” kit can detect up to 28 plasmid copies/µL. Robustness tests showed consistent results, with both contamination levels and the matrices used giving reproducible results. Furthermore, real samples were evaluated to compare the performance of the two methods. The LAMP kit “*Legionella pneumophila* Glow” proved a useful option for the rapid, efficient, and labor-saving screening of different typologies of water samples, offering significant advantages over the traditional method, as it is characterized by a high sensitivity, ease of use for laboratory testing, and a large reduction in analysis time, making it an asset to official controls.

## 1. Introduction

*L. pneumophila* is a waterborne opportunistic pathogen, causing severe pneumonia called Legionnaires’ disease. Among all the species, *L. pneumophila* serogroup 1 is the causative agent of at least 70% of all legionellosis cases in the United States and Europe, making it the most clinically relevant [1]. It is found ubiquitously in natural freshwater environments, and in man-made water systems where it is able to survive within biofilms and intracellularly, in free-living amoebae. Indeed, the role of biofilms is being increasingly recognized as relevant for the establishment and maintenance of a chronic colonization within water distribution and plumbing systems, eluding disinfection treatments [2]. It has also been observed that high percentages of *Legionella* populations in water systems resist in a viable but non-culturable state (VNBC), despite still being infectious [3]. Furthermore, surveillance of Legionnaires’ disease has shown an increasing trend in illness incidence in Europe from 2011 to 2015 [4], which may have been potentially influenced by changes in temperature and rainfall rates due to climate change [5].

Up to 70% of outbreaks are observed in community establishments [6], at healthcare facilities and prisons, even though *L. pneumophila* infections have been generally underestimated because of a lack of clinical awareness and variances in diagnostic procedures [7,8]. The available cultural method requires more than a week to determine both positive and negative results and is laborious and time-consuming. In addition, it does not detect viable but non-culturable bacteria, consequently increasing the risk of false negative results. Molecular methods are more rapid and have an interesting potential for screening, revealing a preliminary result, even if the cultural method remains essential to isolate the strain for further characterization. Since 2015, molecular methods such as real-time PCR were already introduced, and an official reference states that the “Italian guidelines for the prevention and control of legionellosis” aim to obtain the rapid identification of negative samples. Today, PCR is poorly used, both because it detects live and dead bacteria, and because, based on the previous ISO [9], it requires the collection of two liters of water, one liter for molecular testing and the other for cultivation in case of positive molecular results. Recently, a quicker and easier molecular method has been proposed: loop-mediated isothermal amplification (LAMP). In this study, a *L. pneumophila* LAMP assay was validated. LAMP is an innovative specific and cost-effective nucleic acid amplification method for bacterial detection and identification. This novel method is made up of six primers which specifically recognize eight different regions on the target gene [10].

LAMP shows many advantages compared to PCR and real-time PCR as it does not require complex and expensive instruments for carrying out the analysis. In addition, the system validated in this study allows for the automatic interpretation of the results and it is characterized by ready-to-use reagents that simplify the workflow of the analysis which can be carried out by semi-skilled staff [11]. Moreover, LAMP detection sensitivity is 10–100-fold more sensitive and displays a higher amplification efficiency, with higher amounts of amplification products than PCR [12,13,14]. The enzyme involved in the LAMP amplification process, Bst DNA polymerase, allows for strand displacement and amplification under isothermal conditions, and it is more tolerant to PCR-interfering substances such as hemin and anticoagulants [14]. For these reasons, it may represent an ideal candidate for point-of-care diagnosis and when rapid results are needed, such as in the case of *L. pneumophila* facility contamination [15].

This study aimed to carry out a validation of a new commercial system, using the LAMP method, for the detection and quantification of *L. pneumophila* in water samples, according to the ISO [9].

## 2. Materials and Methods

### 2.1. Study Design

The kit “*Legionella pneumophila* Glow” (Enbiotech srl, Palermo, Italy) has been under validation in compliance with ISO [9] “Water quality—Detection and quantification of Legionella spp. and/or *Legionella pneumophila* by concentration and genic amplification by quantitative polymerase chain reaction (qPCR)”. The validation was carried out between March and December 2018 at three Regional Reference Laboratories for the control of legionellosis in Sicily, in Palermo, Catania, and Messina.

The kit “*Legionella pneumophila* Glow” (Enbiotech Cat. No. EBT621) includes rapid preliminary DNA extraction from membrane filters (PES, 0.45 μm, 47 mm, PALL) after 1 L water filtration, genetic amplification using LAMP technology, and the detection of the results using the dedicated device ICGENE mini (Enbiotech Cat. No. EBT801). The kit is composed of a DNA extraction buffer, acting through chemical lysis and all ready-to-use amplification reagents, such as the master mix, mineral oil, tubes strip containing lyophilized primers, and controls.

Therefore, different parameters were analyzed accordingly to the ISO. In particular, the DNA from 1–15 serogroups of *L. pneumophila* was tested (inclusivity), and for exclusivity, the DNA from 18 microorganisms not belonging to the species *L. pneumophila* or the genus *Legionella* spp. was tested, as specified in Table 1. Some strains among those tested were isolated from clinical samples, identified, and confirmed by biochemical identification (Phoenix, BD).

### 2.2. Bacterial Growth Conditions

A total of 25 *Legionella* spp. strains were supplied by the DSMZ (Braunschweig, Germany) and ATCC (American Type Culture Collection, Manassas, VA, USA), and rehydrated, collected, and maintained in culture from the laboratory of the Department of Health Promotion, Mother and Child Care, Internal Medicine and Medical Specialties (University of Palermo), as recommended ISO [9].

Briefly, the 29 strains included in the exclusivity test were chosen based on their presence in the same ecological niche and/or phylogenetic correlation, according to Olabarria et al. [16]. All 25 *Legionella* spp. strains used in this study (Table 1) were rehydrated in a nutrient broth and cultured on a *Legionella* CYE agar base supplemented with L-cysteine HCl (Oxoid Ltd., Hampshire, UK) incubated at 35 ± 2 °C, 3% CO_2_ for 5 days and examined for atypical presence of *Legionella* colonies. Nineteen non-Legionella bacteria were rehydrated in a nutrient broth and cultured according to their appropriate growth agar media and temperature.

*L. pneumophila* and *Legionella* spp. strains were maintained on cryogenic beads at −20 °C; before use, the beads were placed on buffered charcoal yeast extract (BCYE) plates and incubated at 37 °C for 2–5 days in humidified atmosphere (air with 5% CO_2_). Other bacterial strains were placed in tryptone soy agar (TSA) or Columbia blood agar (CBA), incubated at 37 °C for 18–24 h and maintained at 4 °C for two weeks.

### 2.3. Sensitivity and Robustness

Sensitivity was established through serial dilutions of *L. pneumophila* plasmid (DSM 27564) DNA, starting from 1 ng/μL. The last positive dilution was tested with 30 independently laced replicates. Of the replicates, 90% had to be positive to establish the limit of detection (LOD).

In addition, the method robustness was also studied. It consisted of 10 independent samples for three different matrices at two levels of contamination, evaluating whether the detection of the pathogen is not affected by the matrix tested. The following types of matrices were analyzed according to AFNOR NF148 (2015): mineral water, domestic hot water, and cooling tower water. Analyses were carried out under intra-laboratory reproducibility conditions (different days and operators), with a blank sample in addition.

### 2.4. Types of Water Samples

Hence, in total, 60 water samples and 15 blank samples were tested. Firstly, these matrices were tested free from *Legionella* spp. and were then artificially spiked, reaching a final contamination of 105 and 107 CFU/L of *L. pneumophila* (DSM 27564). Lastly, a performance comparison between LAMP and the reference method provided by the ISO [17] was also carried out. Specifically, 72 samples were tested using both methods.

## 3. Results

### 3.1. Inclusivity and Exclusivity

As well as inclusivity and exclusivity, all 15 *L. pneumophila* serogroups showed amplification. The other species tested for exclusivity were negative, except for *L. parisiensis* and *L. tucsonensis*, which showed DNA amplification, as listed in Table 1.

The last positive dilution was 10^−7^ (i.e., 0.0000001 ng/μL) and 27 replicates out of 30 showed amplifications, respecting the 90% confidence interval. Sensitivity was obtained through the following equation, based on the average weight of a base pair (bp) of 650 Daltons (Copy number calculator [Internet]. Rhode Island; available online: http://sciprim.com/html/copyNumb.v2.0.html, accessed on 20 April 2024):

$$\mathrm{Number}\;\mathrm{of}\;\mathrm{copies}=\frac{ng\times (6.022\times 10^{23})}{kb\times (\;660\times 10^{9})}$$

ng = the amount of DNA (in ng) of the last dilution positive for the tested method
6.022 × 10^23^ = Avogadro’s number
kb = plasmid kilobases
660 = mean weight of a nucleobase (dalton)
1 × 10^9^ = conversion factor to ng

Thus, the lowest number of copies detected by the LAMP method is 28 plasmid copies/μL.

### 3.2. Robustness (or Strength)

The results proved 100% of robustness (or strength). The positive samples detected at both contamination levels, 10^5^ and 10^7^ CFU/mL, showed confirmed reproducibility, as shown in Table 2.

### 3.3. Sensitivity and Specificity

In the comparative study, the cultural method and “*Legionella pneumophila* Glow” obtained the same results, showing full agreement: out of 72 samples, 16 were confirmed positive and 56 were negative. The relative accuracy (AC), relative specificity (SP), and sensitivity (SE) were obtained as follows:

AC = (TP + TN)/(PA + NA + FN + FP) × 100%

SP = (TN)/(TN + FP) × 100%

SE = (TP)/(TP + FN) × 100%

TP = true positive samples; TN = true negative samples; FN = false negative samples; and FP = false positive samples (Table 3).

## 4. Discussion

*L. pneumophila* investigation requires rapid isolation and identification to prevent potential infections. A rapid response time could help diagnose and treat water for warding off outbreak events. Thus, the use of alternative methods that rapidly identify the source of contamination is important. In the case of *Legionella* infection, fast identification is mandatory considering its slow-growing bacteria sources that could reach areas or facilities with high population densities. In addition, cultural methods may misdiagnose VBNC cells, which are common environmental forms of *Legionella*.

In this study, all the criteria and parameters tested were achieved according to the ISO requirements, with 100% inclusivity and robustness. The specificity was set at 92%. *Legionella* species such as *L. parisiensis* and *L. tucsonensis* that are rarely isolated showed amplification. *L. parisiensis* was isolated by [18]; only two human isolations have been reported: a liver transplant patient with pneumonia in France [19] and an immunosuppressed patient in Germany [20]. Otherwise, *L. tucsonensis* was isolated from a pleural fluid specimen from an immunosuppressed patient [21]; after that, it has never been sampled, despite environmental and clinical surveillance [22].

Therefore, the obtained data support the suitability of this kit for water matrices. In particular, “*Legionella pneumophila* Glow” proved to be useful for ease of use and significant time reduction in the analytical process. In addition, its analysis time of about 90 min and high sensitivity (28 plasmid copies/μL) may be implemented routinely by competent authorities and laboratories as an effective screening tool for *L. pneumophila*, helping prevent legionnaire.

## 5. Conclusions

The data in this study support the suitability of the “*Legionella pneumophila* Glow” kit for commercial use for different water samples.

This molecular method aims to improve the reliability of the results, making it a promising tool that should be used in addition to cultural analysis. LAMP, as a screening tool, offers a lot of advantages in terms of specificity, sensitivity, and a considerable reduction in time analysis, useful in timely corrective actions. In conclusion, we would like to underline that the investigated method has been validated, showing appropriate performances in accordance with the international standard [9]. Although culturing represents the reference method, as suggested by other authors, PCR should be used simultaneously to culture rather than as an alternative.

## Figures and Tables

**Table 1:** *L. pneumophila* serogroups tested for inclusivity. and strains tested for exclusivity (non-target microorganisms).

Species	Code
*L. pneumophila* serogroup 1	DSM 27564
*L. pneumophila* serogroup 2	DSM 25071
*L. pneumophila* serogroup 3	Not available
*L. pneumophila* subsp. fraseri serogroup 4	ATCC 33156
*L. pneumophila* serogroup 5	DSM 24991
*L. pneumophila* serogroup 6	Not available
*L. pneumophila* serogroup 7	Not available
*L. pneumophila* serogroup 8	Not available
*L. pneumophila* serogroup 9	DSM 25001
*L. pneumophila* serogroup 10	Not available
*L. pneumophila* serogroup 11	DSM 25063
*L. pneumophila* serogroup 12	DSM 25224
*L. pneumophila* serogroup 13	Not available
*L. pneumophila* serogroup 14	Not available
*L. pneumophila* serogroup 15	ATCC 35251
*Legionella longbeachae*	DSM 25315
*Legionella londiniensis*	Not available
*Legionella anisa*	Not available
*Tatlockia micdadei (o Legionella micdadei)*	DSM 16640
*Fluoribacter gormanii (o L. gormanii)*	DSM 25296
*Fluoribacter dumoffii (o L. dumoffii)*	DSM 17625
*Fluoribacter bozemanae (o L.bozemanae o L. bozemanii)*	DSM 16523
*Legionella jordanis*	DSM 19212
*Legionella parisiensis*	DSM 19216
*Legionella tucsonensis*	DSM 19246
*Flavobacterium aquicola*	DSM 100880
*Burkholderia cepacia*	Not available
*Bacillus subtilis*	BCS51
*Aeromonas hydrophila*	ATCC 35650
*Clostridium perfringens*	ATCC 13124
*Listeria monocytogenes*	ATCC 7684
*Staphylococcus aureus*	DSM 2569
*Alcaligenes faecalis*	Not available
*Enterobacter aerogenes*	Not available
*Escherichia coli*	Not available
*Klebsiella oxytoca*	Not available
*Proteus vulgaris*	Not available
*Pseudomonas aeruginosa*	Not available
*Pseudomonas fluorescens*	Not available
*Pseudomonas putida*	Not available
*Serratia marcescens*	Not available
*Stenotrophomonas maltophilia*	Not available
*Enterococcus faecalis*	Not available
*Klebsiella pneumoniae*	Not available

**Table 2:** Robustness of the method with two contamination levels, 10^5^ and 10^7^ CFU/mL. P = positive result, N = negative result.

Matrices	10^5^ CFU/mL (n. Samples)	Results	10^7^ CFU/mL (n. Samples)	Results	Blank Samples (n. Samples)	Results
Control mineral water	10	P	10	P	5	N
Domestic hot water	10	P	10	P	5	N
Cooling tower water	10	P	10	P	5	N
TOT.	30		30		15	

**Table 3:** Relative accuracy, sensitivity, and specificity of the alternative method.

Legionella pneumophila Glow		ISO 11731:2017 [17]	
		Positive (+)	Negative (−)
Water samples	Positive (+)	16	0
	Negative (−)	0	56
	Total samples	99	51

## Data Availability

Data are contained within the article.
